# Supplementary material for: Prediction of Honeydew Contaminations on Cotton Samples by In-Line UV Hyperspectral Imaging
Source: Sensors (Basel). 2022 Dec 28;23(1):319. doi: 10.3390/s23010319 (PMC9823496; doi:10.3390/s23010319)
Supplement: Supplementary file 1 [file sensors-23-00319-s001.zip › sensors-2049639-supplementary - final.pdf]

sensors

MDPI

Supplementary Material

Prediction of Honeydew Contaminations on Cotton Samples by In-Line UV Hyperspectral Imaging

Mohammad Al Ktash[1,2,†], Mona Stefanakis[1,2,†], Frank Wackenhut[1], Volker Jehle[3], Edwin Ostertag[1], Karsten Rebner[1], Marc Brecht[1,2,*]

[1] Center of Process Analysis and Technology (PA&T), School of Applied Chemistry, Reutlingen University, Alteburgstraße 150,72762 Reutlingen, Germany
[2] Institute of Physical and Theoretical Chemistry, Eberhard Karls University Tübingen, Auf der Morgenstelle 18, 72076 Tübingen, Germany
[3] Texoversum Faculty Textile, Reutlingen University, Alteburgstraße 150, 72762 Reutlingen, Germany
* Correspondence: author: marc.brecht@reutlingen-university.de
† These authors contributed equally to the work.

Citation: Al Ktash, M.; Stefanakis, M.; Wackenhut, F.; Jehle, V.; Ostertag, E.; Rebner, K.; Brecht, M. Prediction of Honeydew Contaminations on Cotton Samples by In-Line UV Hyperspectral Imaging. *Sensors* **2023**, *23*,319. https://doi.org/10.3390/s23010319

Academic Editor: Hui-liang Shen

Received: 7 November 2022
Revised: 6 December 2022
Accepted: 13 December 2022
Published: 28 December 2022

Publisher's Note: MDPI stays neutral with regard to jurisdictional claims in published maps and institutional affiliations.

Copyright: © 2022 by the authors. Licensee MDPI, Basel, Switzerland. This article is an open access article distributed under the terms and conditions of the Creative Commons Attribution (CC BY) license (https://creativecommons.org/licenses/by/ 4.0/).

Supplementary Materials:

S1.1 Cotton sample preparation

All details and measured values of the reference sample set are given in Table S1.

Table S1. Description of the cotton sample preparation.

Sample type	Sugar concentration / wt %	Cotton weight / g (± 0.0001 g)	Cotton weight / g after dried at 30 °C, 8 h (± 0.0001 g)	Humidity / % after dried at 30 °C, 8 h	Soaked cotton weight / g after dried at 30 °C, 44 h (± 0.0001 g)	Humidity / % after dried at 30 °C, 44 h	Amount of sugar on cotton / g	Ratio of sugar / g dried cotton / g
A1	2	0.3	0.2879	53.7	0.3734	50.0	0.0855	0.2970
A2	2	0.3	0.2925	53.5	0.3643	50.0	0.0718	0.2455
A3	2	0.3	0.2914	53.3	0.3600	50.0	0.0686	0.2354
B1	1	0.3	0.2917	54.2	0.3295	50.0	0.0378	0.1296
B2	1	0.3	0.2931	54.7	0.3291	50.0	0.0360	0.1228
B3	1	0.3	0.2913	54.2	0.3341	50.0	0.0428	0.1469
C1	0.5	0.3	0.2916	55.2	0.3142	50.0	0.0226	0.0775
C2	0.5	0.3	0.2929	55.6	0.3137	50.0	0.0208	0.0710
C3	0.5	0.3	0.2992	55.7	0.3215	50.0	0.0223	0.0745
D1	0.25	0.3	0.2925	56.0	0.3050	51.0	0.0125	0.0427
D2	0.25	0.3	0.2928	56.2	0.3030	51.0	0.0102	0.0348
D3	0.25	0.3	0.2921	56.2	0.3033	51.0	0.0112	0.0383
E1	0.125	0.3	0.2908	56.5	0.2979	50.5	0.0071	0.0244
E2	0.125	0.3	0.2819	56.9	0.2915	50.0	0.0096	0.0341
E3	0.125	0.3	0.2899	57.2	0.3013	49.9	0.0114	0.0393
F1	0.0625	0.3	0.2903	57.4	0.2997	50.7	0.0094	0.0324
F2	0.0625	0.3	0.2899	57.6	0.2990	49.9	0.0091	0.0314
F3	0.0625	0.3	0.2823	57.7	0.2915	49.7	0.0093	0.0328
CLN1	0	0.3	0.2984	57.7	-	-	-	-
CLN2	0	0.3	0.2991	57.9	-	-	-	-
CLN3	0	0.3	0.2894	58.1	-	-	-	-

S1.2 Additional figures of the principal component analysis of the sugar cotton samples

For the PCA model of the cotton samples with different concentrations of sugar four PCs are necessary. The variance on PC3 is not necessary to distinguish between different sugar concentrations. The information on PC3 might be related to the morphology of the fiber itself. PC1 against PC2, PC3 and PC4 are shown respectively in Figure S1 to complement the PCA sugar model.

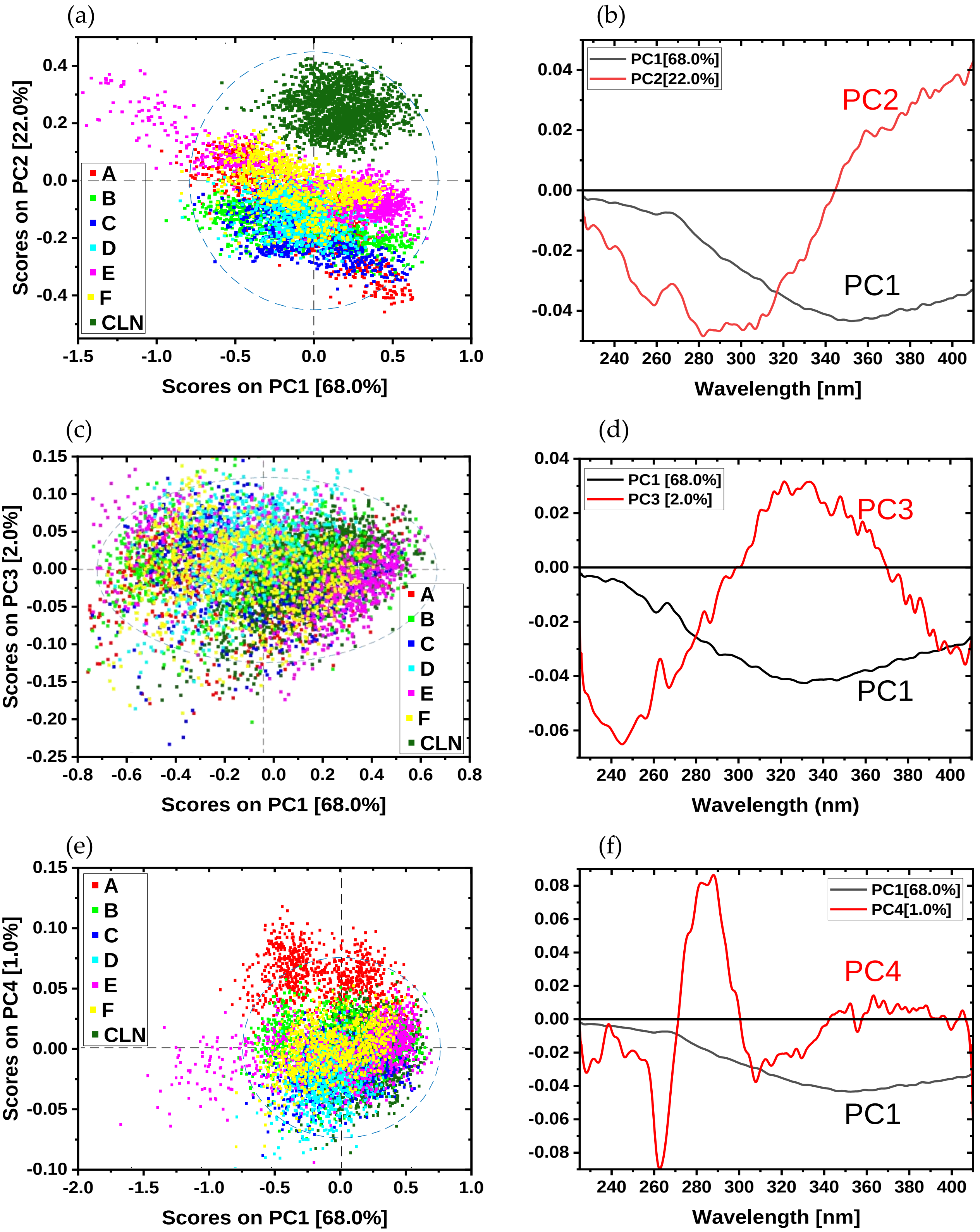

Figure S1. PCA sugar model for the cotton samples with (a,c,e) scores and (b,d,f) corresponding loadings (PC1 black, PC2, PC3 and PC4 red).

S1.3 Pure dried protein spectrum

Protein spectra were acquired to identify the information in the range of 250 nm to 280 nm. The protein was solved in distilled water and the solution was dropped on a piece of PTFE. Afterwards, the sample was dried in a vacuum oven (see 2.2). Data was acquired with the hyperspectral imaging setup with the settings mentioned in 2.3 and 2.4. This experiment was necessary to verify the spectral range between 250 nm and 280 nm contains true information and is not an artifact due to the efficiency of the detector and the weak intensity light source in the UV range [1].

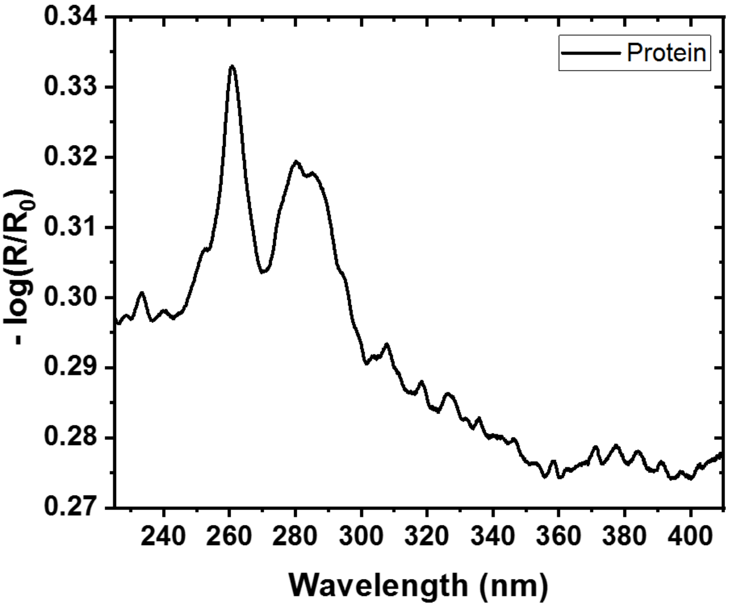

Figure S2. Mean spectrum of pure dried protein on PTFE.

S1.3 X-loadings weights and x-loadings of the PLS-R model

For model building and understanding the PLS-R factor loadings and loading weights for all five factors are dispayed in the Figure S3.

a) b)

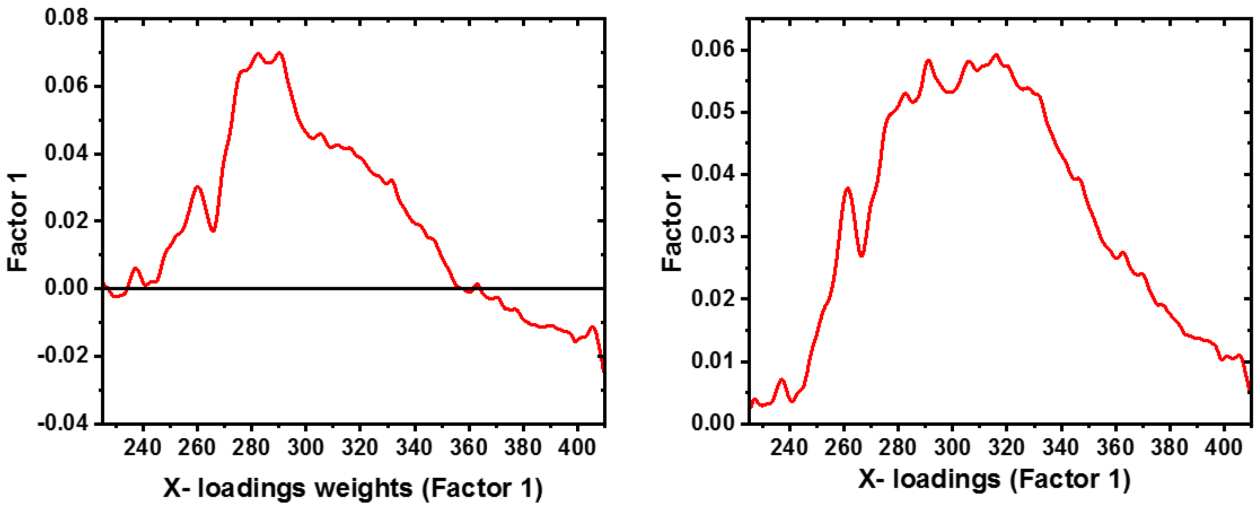

c)

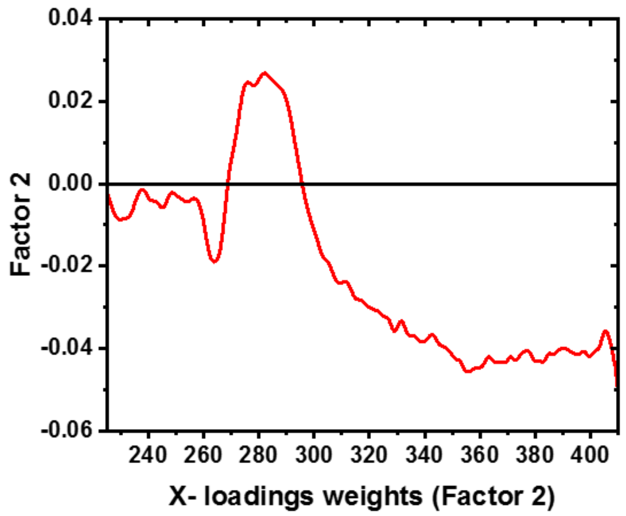
0.04
0.02
0.00
-0.02
-0.04
-0.06
Factor 2
240 260 280 300 320 340 360 380 400
X- loadings weights (Factor 2)

d)

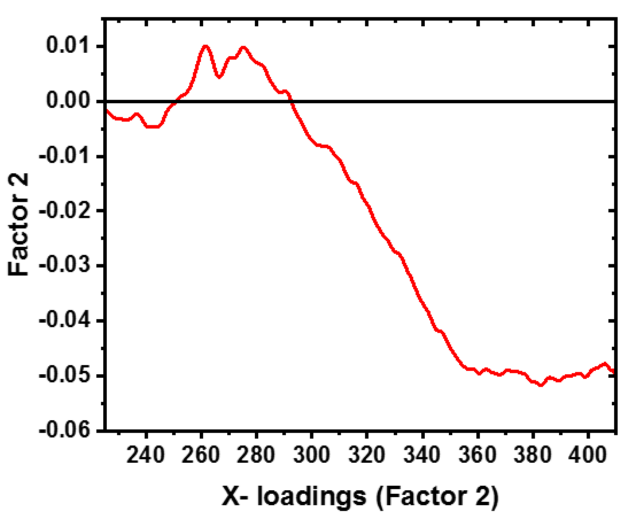
0.01
0.00
-0.01
-0.02
-0.03
-0.04
-0.05
-0.06
Factor 2
240 260 280 300 320 340 360 380 400
X- loadings (Factor 2)

e)

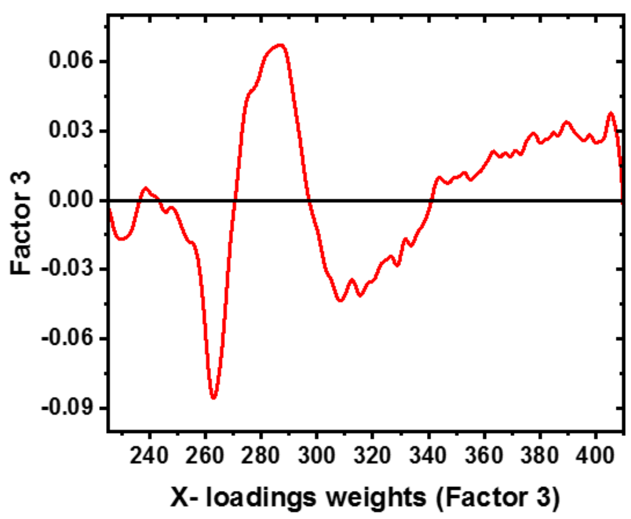
0.06
0.03
0.00
-0.03
-0.06
-0.09
Factor 3
240 260 280 300 320 340 360 380 400
X- loadings weights (Factor 3)

f)

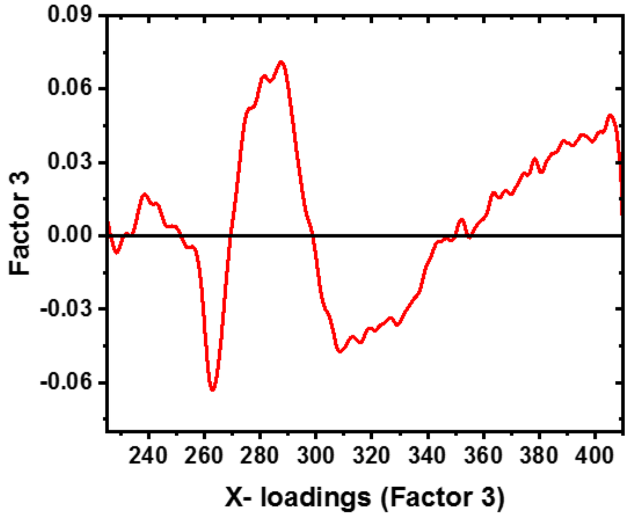
0.09
0.06
0.03
0.00
-0.03
-0.06
Factor 3
240 260 280 300 320 340 360 380 400
X- loadings (Factor 3)

g)

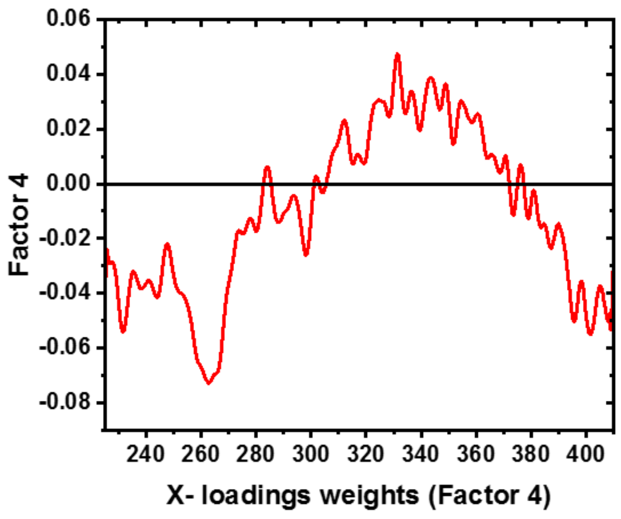
0.06
0.04
0.02
0.00
-0.02
-0.04
-0.06
-0.08
Factor 4
240 260 280 300 320 340 360 380 400
X- loadings weights (Factor 4)

h)

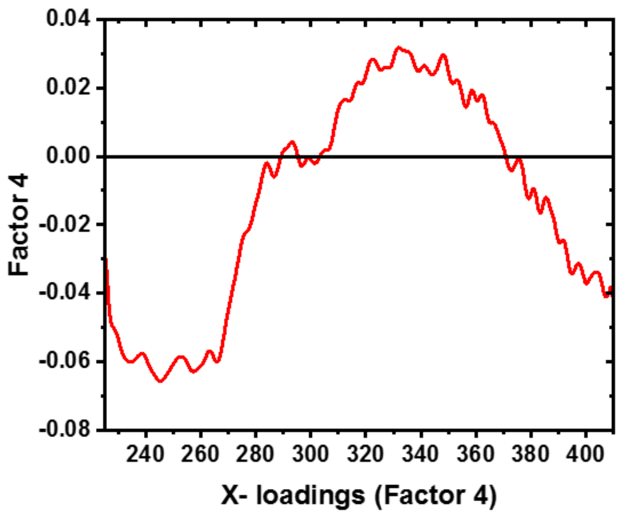
0.04
0.02
0.00
-0.02
-0.04
-0.06
-0.08
Factor 4
240 260 280 300 320 340 360 380 400
X- loadings (Factor 4)

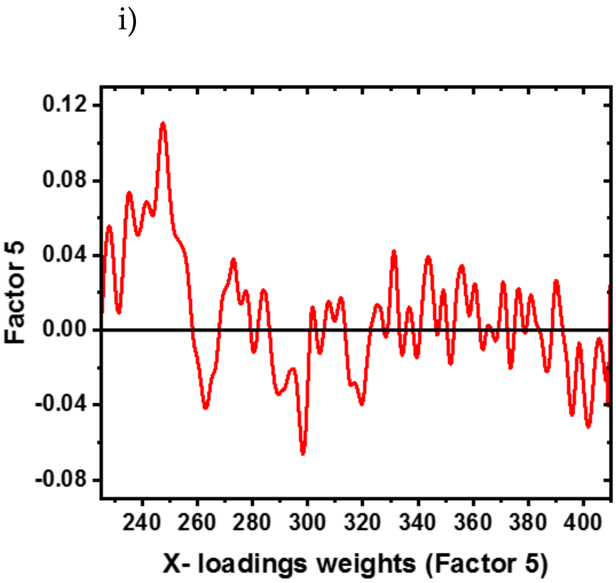

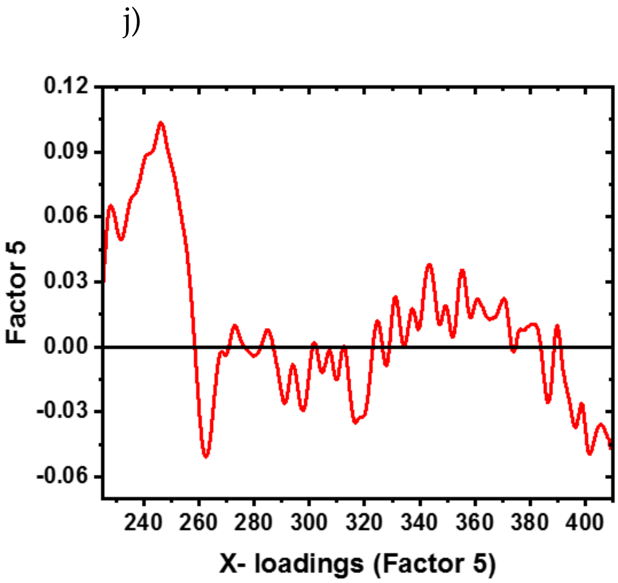

Figure S3. X-loadings weights and x-loadings for factor 1 (a, b), factor 2 (c, d), factor 3 (e, f), factor 4 (g, h) and factor 5 (i, j), respectively.

S1.4 References

1. Al Ktash, M.; Stefanakis, M.; Boldrini, B.; Ostertag, E.; Brecht, M. Characterization of pharmaceutical tablets using UV hyperspectral imaging as a rapid in-line analysis tool. *Sensors* **2021**, *21*, 4436, doi:10.3390/s21134436.
